# Nitrogen Supply Mitigates Temperature Stress Effects on Rice Photosynthetic Nitrogen Use Efficiency and Water Relations

**DOI:** 10.3390/plants14060961

**Published:** 2025-03-19

**Authors:** Zhuang Xiong, Fangzhou Zheng, Chao Wu, Hui Tang, Dongliang Xiong, Kehui Cui, Shaobing Peng, Jianliang Huang

**Affiliations:** 1 Guangxi Key Laboratory of Plant Functional Phytochemicals and Sustainable Utilization, Guangxi Institute of Botany, Guangxi Zhuang Autonomous Region and Chinese Academy of Sciences, Guilin 541006, China; xiongzhuang@gxib.cn (Z.X.); wuchao@gxib.cn (C.W.); th@gxib.cn (H.T.); 2 State Key Laboratory of Plant Physiology and Biochemistry, College of Life Sciences, Zhejiang University, Hangzhou 310058, China; zkana@163.com; 3 National Key Laboratory of Crop Genetic Improvement, Ministry of Agriculture Key Laboratory of Crop Ecophysiology and Farming System in the Middle Reaches of the Yangtze River, College of Plant Science and Technology, Huazhong Agricultural University, Wuhan 430070, China; dlxiong@mail.hzau.edu.cn (D.X.); cuikehui@mail.hzau.edu.cn (K.C.); speng@mail.hzau.edu.cn (S.P.)

**Keywords:** nitrogen, temperature, photosynthesis, photosynthetic nitrogen use efficiency, intrinsic water use efficiency

## Abstract

Climate-change-induced temperature fluctuations pose significant threats to global rice production, particularly through their impact on photosynthetic efficiency. The differential mechanisms by which low and high temperatures affect leaf photosynthetic processes in rice remain poorly understood. Here, we investigate the effects of temperature stress (15 °C, 30 °C, 45 °C) on rice photosynthetic performance across a gradient of nitrogen supply levels: low nitrogen (LN), medium nitrogen (MN), and high nitrogen (HN). The low temperature exhibited stronger negative impacts on photosynthesis than the high temperature, primarily through increased mesophyll limitation and disrupted cellular CO_2_ diffusion, while the high temperature showed less pronounced effects, particularly under HN and MN conditions. While photosynthetic nitrogen use efficiency (PNUE) decreased with increasing nitrogen under the optimal temperature, moderate nitrogen supply maintained optimal PNUE under temperature stress, suggesting that a balanced nitrogen level is crucial for maximizing both photosynthetic capacity and nitrogen use efficiency. Plants with adequate nitrogen maintained higher intrinsic water use efficiency (_i_*WUE*) under both temperature extremes through improved coordination between CO_2_ uptake and water loss. Our findings reveal distinct mechanisms underlying low- and high-temperature stress effects on photosynthesis and highlight the importance of optimizing nitrogen management for enhancing crop resilience to temperature extremes under climate change.

## 1. Introduction

Climate change has emerged as one of the most pressing challenges facing global agriculture and is characterized by increasing frequency and intensity of extreme weather events, particularly temperature fluctuations [1,2]. Rice, as one of the most important food crops, feeding more than half of the population worldwide, is particularly vulnerable to temperature stress, as it is cultivated year-round in various climatic conditions where both cold and heat stress can substantially impact its growth and yield [3,4,5]. Low-temperature stress, especially during the early growing season, can severely inhibit seedling establishment and early growth, while high-temperature stress during reproductive stages can lead to reduced grain filling and yield losses [6,7]. The increasing unpredictability of temperature patterns due to climate change further complicates crop management strategies and highlights the urgent need to understand and enhance crop resilience to extreme temperature events. Photosynthesis, the fundamental process underlying biomass accumulation and yield formation, is highly sensitive to temperature fluctuations [8,9]. Temperature extremes can affect photosynthetic efficiency through multiple mechanisms, including stomatal conductance (*g*_s_), mesophyll conductance (*g*_m_), and biochemical capacity to fix carbon [10,11]. The optimization of photosynthetic performance under varying temperature conditions is, therefore, crucial for maintaining crop productivity in the face of climate change. However, our understanding of the differential impacts of low- and high-temperature stress on photosynthetic processes, and the mechanisms by which plants can maintain photosynthetic efficiency under both conditions, remains incomplete.

Nitrogen (N) plays a pivotal role in plant photosynthetic capacity and stress tolerance. As a key component of proteins, enzymes, and chlorophyll, adequate nitrogen nutrition is essential for maintaining optimal photosynthetic machinery and stress response mechanisms [12,13]. In the photosynthetic apparatus, nitrogen is a crucial constituent of various components, including light-harvesting complexes, electron transport chain proteins, and, particularly, Rubisco, which accounts for a substantial portion of leaf nitrogen content [14]. Nitrogen deficiency can significantly impair photosynthetic capacity by reducing chlorophyll content, decreasing Rubisco activity, and limiting electron transport capacity [15]. Photosynthetic nitrogen use efficiency (PNUE), which reflects the effectiveness of nitrogen allocation for photosynthetic function, is a critical parameter in understanding the relationship between nitrogen investment and photosynthetic performance [16]. PNUE represents the carbon fixed per unit of leaf nitrogen and serves as an important indicator of plant resource use strategy and adaptation to environmental conditions [12,17,18]. Under normal conditions, PNUE typically shows a negative correlation with leaf nitrogen content, suggesting diminishing returns on nitrogen investment, which reflects the complex balance between nitrogen allocation to different photosynthetic components and the optimization of resource use efficiency [19,20,21]. The regulatory interaction between nitrogen nutrition and temperature stress on photosynthetic performance involves complex biochemical and physiological mechanisms which may potentially limit the effective use of nitrogen-containing compounds and disrupt the typical PNUE–nitrogen-content relationship. This is particularly relevant in the context of climate change, where crops need to maintain both high productivity and resource use efficiency under increasingly variable temperature conditions.

Intrinsic water use efficiency (_i_*WUE*), defined as the ratio of photosynthetic carbon assimilation to stomatal conductance, represents a critical physiological trait integrating plant carbon and water relations [22,23]. The effects of nitrogen fertilization on leaf _i_*WUE* remain controversial, with studies reporting both positive and negative responses under various environmental conditions. An enhanced nitrogen supply typically increases stomatal conductance more proportionally than photosynthetic capacity, therefore leading to improved iWUE [24]. However, Querejeta et al. (2022) indicated that higher leaf nitrogen content is linked to both higher carboxylation capacity and tight stomatal regulation of transportation for dryland trees, allowing them to achieve higher _i_*WUE* [25]. Under conditions of constant atmospheric water content, temperature elevation leads to exponential increases in saturation vapor pressure, following the Clausius–Clapeyron relationship. This temperature-induced increases in vapor pressure deficit (VPD) trigger stomatal closure, consequently limiting photosynthetic carbon assimilation (*A*) through reduced intercellular CO_2_ concentration (C_i_) [9,26]. The temperature dependency of the net photosynthetic rate (*A*) represents the integrated response of multiple physiological processes, including maximum Rubisco carboxylation capacity (*V*_cmax_) and maximum electron transport rate (*J*_max_), coupled with CO_2_ concentration inside the leaf that is regulated by diffusional conductance (*g*_s,_ *g*_m_) [27,28,29]. These processes lead to increased complexity in the interactive effects of nitrogen fertilizer and temperature on _i_*WUE*. Understanding these interactions is crucial for predicting plant physiological responses to climate change and optimizing nitrogen fertilization strategies.

To address these knowledge gaps, we conduct an experiment to investigate the effects of low (15 °C), optimal (30 °C), and high (45 °C) temperature treatments on photosynthetic parameters, photosynthetic nitrogen use efficiency (PNUE), and intrinsic water use efficiency (_i_*WUE*) under contrasting nitrogen supplies (low, medium, and high) in rice. Our objectives are to (1) elucidate the differential impacts and underlying mechanisms of low- and high-temperature stress on photosynthetic processes, (2) examine the relationship between nitrogen supply and PNUE under temperature stress, and (3) explore the interactive effects of nitrogen supply and temperature stress on leaf _i_*WUE*.

## 2. Materials and Methods

### 2.1. Plant Growth

The rice cultivar Huanghuazhan (HHZ) was sown in holy plates filled with soil under open-air conditions on the campus of Huazhong Agricultural University in October 2021. Twenty-five days after sowing, seedlings were transplanted into 10 L pots containing 10 kg of crushed dry field paddy soil. Plants were maintained in a growth chamber under controlled environmental conditions with a 12 h light (30 °C) and 12 h dark (25 °C) cycle and photosynthetically active radiation (PAR) of 400 μmol m⁻^2^ s⁻^1^ at the soil surface. Nitrogen fertilization treatments comprised three levels: 0 (low nitrogen; LN), 1.2 (middle nitrogen; MN), and 3.6 g (high nitrogen; HN) N pot⁻^1^ [24]. Nitrogen was applied as urea in three split applications following a 4:3:3 ratio at basal, tillering, and panicle initiation stages. Additionally, 1.5 g each of phosphorus and potassium were incorporated into each pot as basal fertilizer in the form of superphosphate and potassium chloride, respectively. Each treatment consisted of four replicate pots with three plants per pot. All measurements were conducted thirty-five days after transplanting.

### 2.2. Leaf Gas Exchange Measurements

Gas exchange measurements were performed on the youngest fully expanded leaves using a Li-6800 portable photosynthesis system (LI-COR). Environmental conditions during measurements were maintained at three temperature levels (15 °C, 30 °C, 45 °C) in the growth chamber, with photosynthetic photon flux density (PPFD) at 400 μmol m⁻^2^ s⁻^1^ and relative humidity at 75%. *A/C*_i_ curves were constructed to evaluate temperature responses of leaf gas exchange. Leaves were initially acclimated at 1500 μmol m⁻^2^ s⁻^1^ PPFD and 400 ppm CO_2_ until *A* and *g*_s_ stabilized. Subsequently, leaves were exposed to a sequence of CO_2_ concentrations (400, 300, 200, 150, 100, 50, 400, 600, 800, 1000, 1200, 1500, and 1800 ppm), with measurements recorded after achieving steady-state conditions (approximately 4 to 6 min for each step).

### 2.3. Agronomical Traits

Following completion of gas exchange measurements, the youngest fully expanded leaves were sampled for nitrogen (N) content determination. Photosynthetic nitrogen use efficiency (PNUE) was calculated as the ratio of photosynthetic rate (*A*) to leaf nitrogen content per unit area (N_area_) [30]. Plant growth parameters were assessed using one randomly selected plant from each pot. Total leaf area was quantified using a Li-3000 leaf area meter (LI-COR Inc., Lincoln, NE, USA). Plants were separated into leaves and stems, and tiller numbers per plant were recorded. All plant materials were subsequently oven-dried at 80 °C until a constant weight was reached for determination of total aboveground biomass.

### 2.4. Calculation

During *A/C*_i_ curve measurements, steady-state fluorescence (*F_s_*) and maximum fluorescence (*F_m_*^′^) were recorded at a CO_2_ concentration of 400 ppm. The actual photosynthetic efficiency of photosystem II (Φ_PSII_) was calculated using the following equation:

ΦPSII=Fm′−FsFm′

Electron transport rate (*J*) was subsequently determined as follows:

$$J={\;\Phi }_{\mathrm{PSII}}\times PPFD\times \alpha \beta$$

where *α* represents the leaf absorptance and *β* represents the partitioning of absorbed quanta between PSI and PSII.

The chloroplast CO_2_ concentration (*C*_c_) and mesophyll conductance (*g*_m_) were calculated using the following equations:

$$C_{c}=\frac{\Gamma^{*\;}\times \left(J+8\left(A+R_{d}\right)\right)}{J-4\left(A+R_{d}\right)}\;g_{m}=\frac{A}{C_{i}-C_{c}}$$

where *C*_i_ represents intercellular CO_2_ concentration, Γ* denotes the CO_2_ compensation point in the absence of mitochondrial respiration (40 μmol mol⁻^1^), and *R*_d_ represents day respiration (1 μmol m⁻^2^ s⁻^1^) for rice leaves, following established values from previous studies [31,32].

Relative photosynthetic limitations under constant light conditions, including stomatal (*ls*), mesophyll (*lm*), and biochemical (*lb*) limitations, were calculated following the methods of [33]:

$$ls=\frac{g_{t}/g_{s}\times \partial A/\partial C_{c}}{g_{t}+\partial A/\partial C_{c}}\;lm=\frac{g_{t}/g_{m}\times \partial A/\partial C_{c}}{g_{t}+\partial A/\partial C_{c}}\;lb=\frac{g_{t}}{g_{t}+\partial A/\partial C_{c}}$$

where *g_t_* represents the total CO_2_ diffusion conductance of *g_s_* and *g_m_*, calculated as $g_{t}=1/\left(1/g_{s}+1/g_{m}\right)$, and $\partial A/\partial C_{c}$ is the slope of the *A* vs. *C_c_* response curve.

### 2.5. Statistical Analysis

Statistical analyses were performed using SPSS 21.0 (SPSS for Windows, Chicago, IL, USA) and GraphPad Prism 10. One-way ANOVAs and two-way ANOVAs were analyzed by using the Tukey HSD test for comparisons of means, following verification of data normality and homogeneity of variance. Linear regression analysis was performed using SigmaPlot 12.5 (Systat Software Inc., San Jose, CA, USA) to evaluate relationships among the measured parameters.

## 3. Results

### 3.1. Effect of N Supplies on Leaf Photosynthesis and Plant Growth

**Figure 1:**
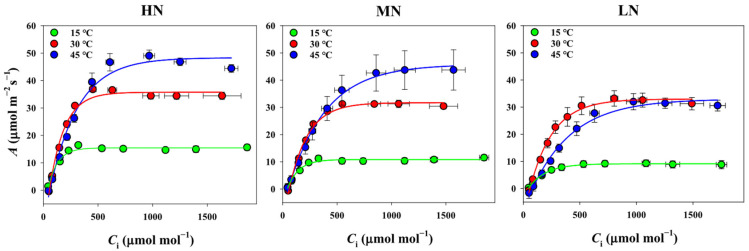
Impact of temperature stress (15 °C and 45 °C) on the photosynthetic CO₂ response curves across varying nitrogen (N) treatments (HN, MN, LN). *A* and *C*_i_ are the photosynthetic rate and the intercellular CO_2_ concentration, respectively. Each data point represents the mean ± standard deviation (SD) derived from four independent biological replicates.

The investigation of nitrogen supply effects on leaf gas exchange parameters revealed that the photosynthetic rate (*A*) at a CO_2_ concentration of 400 μmol mol⁻^1^ significantly increased with higher nitrogen supplies across all temperature treatments (Figure 1). Stomatal conductance (*g*_s_) showed significant differences among nitrogen supplies at 15 °C and 30 °C, but not at 45 °C (Table 1). Intrinsic water use efficiency (_i_*WUE*) exhibited no significant differences among the nitrogen treatments at 30 °C, whereas _i_*WUE* in the LN treatment was markedly lower than that in the HN and MN treatments at both 15 °C and 45 °C. While the intercellular CO_2_ concentration (*C*_i_) remained constant across nitrogen treatments, mesophyll conductance increased significantly with higher nitrogen supplies across all temperature treatments, subsequently resulting in elevated the chloroplast CO_2_ concentrations (*C*_c_) (Table 1). Additionally, the electron transport rate exhibited significant differences among nitrogen treatments, indicating that both diffusional and biochemical processes were influenced by nitrogen supply levels.

Leaf nitrogen content and whole-plant agronomic characteristics varied significantly with nitrogen treatment (Table 2). Leaf nitrogen content showed marked differences based on both leaf weight (N_mass_, 17.6–37.8 mg g⁻^1^) and leaf area (N_area_, 1.02–2.13 g m⁻^2^) across nitrogen treatments. While leaf mass per area (LMA) showed no clear response to nitrogen supply, whole-plant agronomic characteristics demonstrated significant increases with enhanced nitrogen supply: total leaf area increased from 0.28 to 1.79 × 10^3^ cm^2^ plant⁻^1^, tiller number increased from 3.5 to 11.5 plant⁻^1^, plant height increased from 84 to 114 cm, and biomass increased from 4.0 to 22.5 g plant⁻^1^ (Table 2).

### 3.2. Response of Leaf Gas Exchange Parameters to Low and High Temperatures

**Figure 2:**
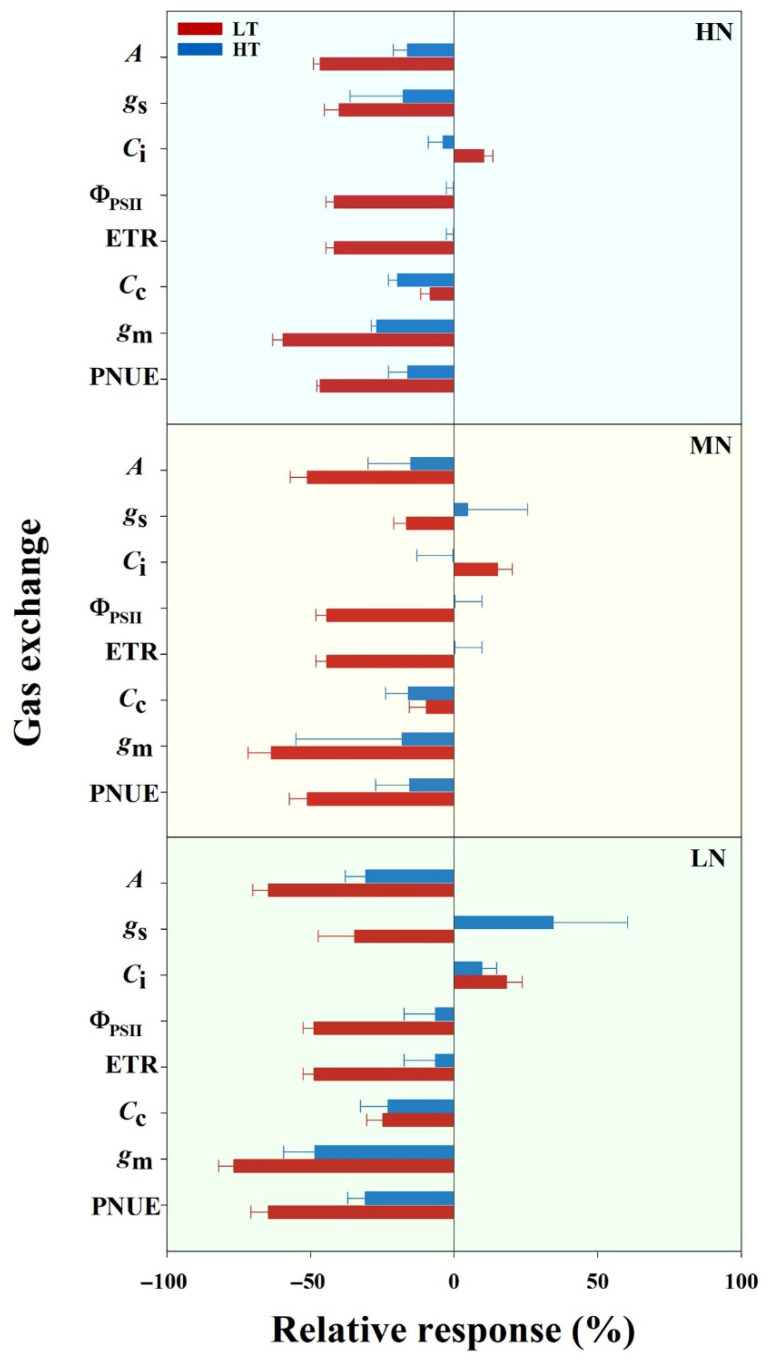
Response of leaf gas exchange parameters to temperature extremes (LT and HT) across N treatments (LN, MN and HN). The red and blue bars indicate low temperature (LT) and high temperature (HT) treatments, respectively. *A*: photosynthetic rate; *g*_s_: stomatal conductance; *C*_i_: intercellular CO_2_ concentration; Φ_PSII_: actual PSII efficiency; ETR: electron transport rate; *C*_c_: chloroplast CO_2_ concentration; *g*_m_: mesophyll conductance; PNUE: photosynthetic nitrogen use efficiency. Values represent means ± standard deviation (SD) with four independent biological replicates.

The assessment of temperature effects on leaf potential photosynthesis through *A/C*_i_ curve analysis at 15 °C, 30 °C, and 45 °C across nitrogen treatments revealed distinct patterns (Figure 1). Under HN and MN supplies, potential photosynthesis was notably higher at 45 °C compared to 30 °C, whereas the LN supply showed minimal differences between these temperatures. The low temperature (15 °C) substantially decreased the potential photosynthesis across all nitrogen treatments (Figure 1). Further analysis of the relative responses of gas exchange parameters to low and high temperature treatments demonstrated variations ranging from −76% to 35% (Figure 2). As expected, leaf gas exchange responses to temperature treatments differed among nitrogen supplies, with photosynthesis under the LN supply exhibiting stronger responses to both low and high temperatures compared to the MN and HN supplies. Notably, leaf gas exchange traits demonstrated a greater sensitivity to low-temperature damage compared to high-temperature stress.

**Figure 3:**
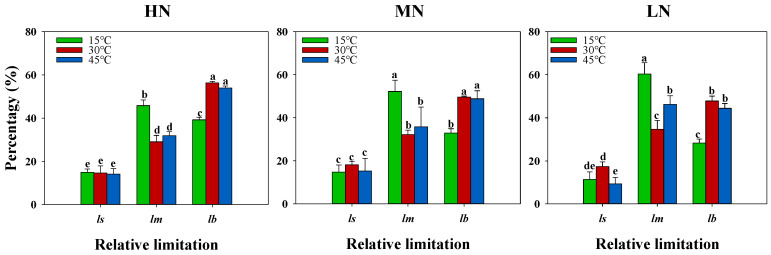
Interactive effects of nitrogen supply and temperature stress on diffusional and biochemical limitations to photosynthesis. Temperature treatments during gas exchange measurements are represented by colored bars: green (15 °C), red (30 °C), and blue (45 °C). ls: stomatal limitation; lm: mesophyll limitation; lb: biochemical limitation. Values are presented as means ± standard deviation (SD) with four biological replicates. Different letters indicate significant differences according to Tukey HSD (0.05).

Analysis of diffusional and biochemical limitations to photosynthesis across nitrogen and temperature treatments revealed that stomatal limitations comprised a smaller fraction of photosynthetic limitations compared to mesophyll and biochemical processes (Figure 3). Under the HN and MN supplies, the high temperature showed minimal effects on diffusional and biochemical limitations, whereas under the LN supply, the high temperature (45 °C) significantly decreased stomatal limitation while increasing mesophyll limitation. The low temperature (15 °C) exhibited contrasting effects, showing minimal impact on stomatal limitation but significantly increasing mesophyll limitation while decreasing biochemical limitation under the HN and MN supplies. Furthermore, the low temperature significantly enhanced mesophyll limitation while reducing both stomatal and biochemical limitations, suggesting that temperature effects on photosynthesis primarily operated through modifications in mesophyll limitation.

### 3.3. Effects of N Supplies on Leaf PNUE Under Low and High Temperatures

**Figure 4:**
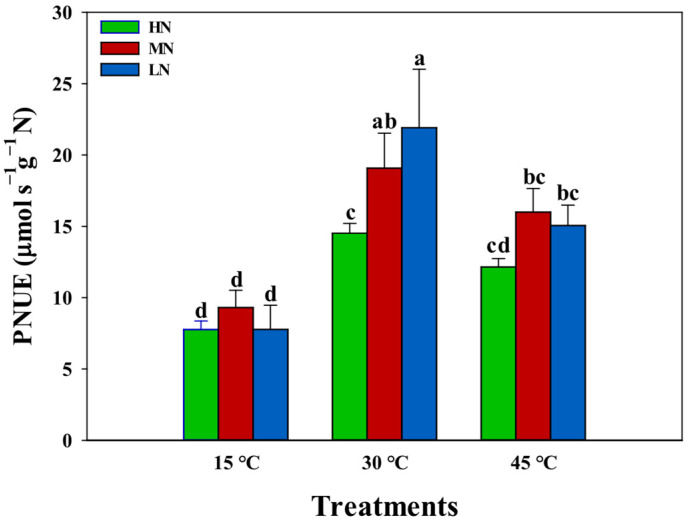
Interactive effects of nitrogen (N) supply and temperature stress on leaf photosynthetic nitrogen use efficiency (PNUE, μmol s^−1^ g^−1^ N). Colored bars represent different N treatment conditions: HN (green), MN (red), and LN (blue). Values are presented as means ± standard deviation (SD) with four biological replicates. Different letters indicate significant differences according to Tukey HSD (0.05).

**Figure 5:**
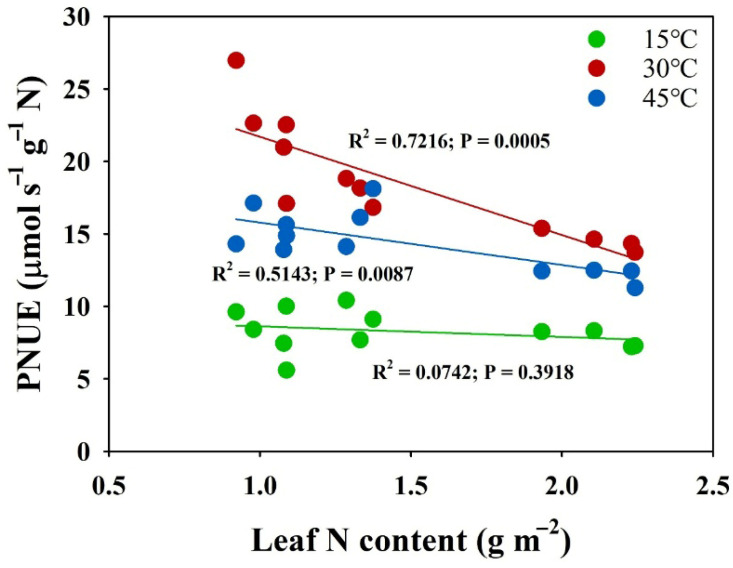
Correlation between photosynthetic nitrogen use efficiency (PNUE, μmol s^−1^ g^−1^ N) and leaf N content (g m^−2^) under three distinct temperature regimes. PNUE represents the rate of carbon assimilation per unit of leaf N content.

The evaluation of nitrogen supply effects on leaf photosynthetic nitrogen use efficiency (PNUE) across temperature treatments revealed distinct temperature-dependent patterns (Figure 4). Under normal temperature conditions (30 °C), leaf PNUE demonstrated a significant decrease with increasing nitrogen supply levels. However, under low-temperature conditions (15 °C), no significant differences in leaf PNUE were observed across nitrogen treatments. Under high-temperature conditions (45 °C), PNUE showed minimal differences between the MN and LN supplies, while the HN treatment exhibited significantly lower PNUE values compared to both the MN and LN supplies. The analysis of the relationship between PNUE and leaf nitrogen content revealed significant negative correlations under both the 30 °C and 45 °C conditions (Figure 5). However, the slope of the PNUE–leaf-nitrogen-content regression was substantially lower at 45 °C compared to 30 °C, indicating temperature-dependent modifications in nitrogen utilization efficiency. Notably, under low-temperature conditions (15 °C), no correlation was observed between PNUE and leaf nitrogen content, suggesting disruption of the typical nitrogen–photosynthesis relationship under cold stress.

## 4. Discussion

### 4.1. Low Temperature Exerts a Greater Negative Impact on Leaf Photosynthesis Through Distinct Mechanistic Pathways

Global climate change induces increasingly frequent and intense extreme weather events, such as high and low temperatures, posing severe threats to rice production [34,35]. Year-round rice cultivation across spring and summer seasons exposes crops to both low- and high-temperature stress, leading to impaired photosynthetic capacity and reduced grain yield. The differential mechanisms by which low and high temperatures affect leaf photosynthetic processes in rice remain poorly understood. In this experiment, our findings revealed a more pronounced impact of the low temperature (15 °C) compared to the high temperature (45 °C) on leaf photosynthetic performance, with responses varying based on nitrogen availability (Figure 2). The relative response of gas exchange parameters showed substantial variations (−76% to 35%), with the low temperature consistently causing more severe reductions in photosynthetic parameters across all nitrogen treatments. This greater sensitivity to low temperatures was particularly evident in LN treatments, where plants exhibited the strongest response to temperature stress (Figure 2). Notably, while plants under the HN and MN treatments maintained or even showed higher potential photosynthesis at 45 °C than at 30 °C, all nitrogen treatments experienced significant decreases in photosynthetic capacity at 15 °C (Figure 1; Table 1). This differential response suggests that plants have more robust mechanisms for coping with heat stress compared to cold stress, particularly when adequately supplied with nitrogen.

The contrasting effects of low and high temperatures on photosynthesis were underpinned by distinct physiological mechanisms. Under the low temperature, we observed a significant increase in mesophyll limitation accompanied by decreased biochemical limitations across all nitrogen treatments (Figure 3), suggesting that cold stress primarily impairs the CO_2_ diffusion pathway at the cellular level, possibly through reduced membrane fluidity and altered cellular organization [36]. In contrast, the high temperature showed minimal effects on both diffusional and biochemical limitations under the HN and MN treatments, only significantly affecting mesophyll conductance under LN conditions (Figure 3). This suggests that an adequate nitrogen supply effectively maintains membrane integrity and cellular function under heat stress [37,38]. Interestingly, stomatal limitations played a relatively minor role in both temperature responses, though the high temperature decreased stomatal limitation particularly under LN conditions (Figure 3). The dominance of mesophyll limitations under low temperature, coupled with the nitrogen-dependent response to high temperature, indicates that these temperature extremes affect photosynthesis through different pathways: cold stress primarily disrupts cellular organization and CO_2_ diffusion, while heat stress effects are more closely tied to nitrogen-dependent protective mechanisms. These mechanistic insights suggest that strategies to enhance temperature stress tolerance should be tailored differently for cold versus heat stress, with particular attention to maintaining cellular organization under low temperature and ensuring adequate nitrogen nutrition for heat stress protection.

### 4.2. Nitrogen Supply Alleviates Temperature-Stress-Induced PNUE Depression

Previous studies have extensively investigated the ameliorative effects of nitrogen on temperature-stress-induced photosynthetic inhibition [39,40]. Also, our results demonstrate that increased nitrogen supply effectively mitigated the adverse effects of both low- and high-temperature stress on photosynthetic capacity (Table 1). Several key findings evidenced this protective effect. First, plants under medium and high nitrogen supplies (HN and MN) exhibited significantly higher photosynthetic rates across all temperature treatments compared to low-nitrogen (LN) conditions. Notably, under the HN and MN treatments, plants maintained higher potential photosynthesis at 45 °C than at 30 °C, while this enhancement was absent under LN conditions, suggesting that adequate nitrogen nutrition is crucial for heat stress tolerance (Figure 1; Table S1). The mechanisms underlying this nitrogen-mediated protection can be attributed to both diffusional and biochemical processes. Our analysis revealed that mesophyll conductance significantly increased with higher N supplies across all temperature treatments, leading to enhanced CO_2_ concentrations in the chloroplasts (Cc) (Table 1). This suggests that adequate nitrogen nutrition improves the CO_2_ diffusion pathway from substomatal cavities to chloroplasts, a process which is crucial for maintaining photosynthetic efficiency under temperature stress [41]. Furthermore, analysis of photosynthetic limitations revealed that stomatal limitations comprised a relatively small fraction compared to mesophyll and biochemical limitations under temperature stress (Figure 3). This finding suggests that the protective effect of nitrogen primarily operates through maintaining mesophyll conductance and biochemical processes rather than through stomatal regulation. The increased mesophyll limitation under low-temperature stress across all N treatments, particularly under LN conditions, indicates that cold stress primarily affects the CO_2_ diffusion pathway at the cellular level.

Optimization of PNUE has emerged as a critical priority, as excessive nitrogen application not only reduces economic efficiency but also causes environmental degradation through non-point source pollution, soil acidification, and greenhouse gas emissions [42,43,44,45]. In the present study, increasing nitrogen supply significantly decreased photosynthetic nitrogen use efficiency (PNUE) at the optimal temperature (30 °C), suggesting a diminishing return on photosynthetic capacity with additional nitrogen investment (Figure 4). This inverse relationship between PNUE and leaf nitrogen content suggests that plants under a higher N supply operate below their maximum nitrogen use efficiency under normal conditions. However, this pattern changed dramatically under temperature stress. Under low temperature (15 °C), the differences in PNUE across nitrogen treatments disappeared, suggesting that cold stress fundamentally altered nitrogen utilization patterns (Figure 4). This could be attributed to the enhancement of photosynthetic efficiency with higher N supplies. Under the high temperature (45 °C), we observed an interesting pattern where PNUE in the HN treatment was significantly lower than that in the MN and LN treatments, while little difference existed between the MN and LN treatments (Figure 4). The lower slope of PNUE-leaf-N-content regression at 15 °C and 45 °C compared to 30 °C indicates that the high temperature altered the efficiency of nitrogen utilization for photosynthesis (Figure 5). This suggests that increased nitrogen supply can help maintain PNUE under temperature stress. Moreover, the moderate nitrogen (MN) supply maintained optimal PNUE under temperature stress, suggesting that there may be an optimal nitrogen supply level that balances photosynthetic performance with nitrogen use efficiency [18]. These findings have important implications for nitrogen management strategies under variable temperature conditions. Future research should focus on identifying these optimal nitrogen levels for different temperature scenarios and understanding the molecular mechanisms underlying the interaction between nitrogen utilization and temperature stress responses.

### 4.3. Combined Effects of Nitrogen Supply and Temperature on Leaf Intrinsic Water Use Efficency (_i_WUE)

Currently, the respective effects of nitrogen application rate and temperature on leaf intrinsic water use efficiency (_i_*WUE*) remain controversial, and the physiological mechanisms underlying their combined regulation of _i_*WUE* are still not well understood [46,47,48,49]. Here, our results revealed complex interactions between temperature stress and nitrogen supply in regulating leaf _i_*WUE* (Table 1). Several key findings emerged from this study that advance our understanding of plant water–carbon relationships under temperature stress conditions. First, the differential responses of _i_*WUE* to temperature stress across nitrogen treatments suggest that nitrogen nutritional status plays a crucial role in modulating plant water use strategies. Under the optimal temperature (30 °C), we observed no significant differences in _i_*WUE* among nitrogen treatments, indicating that plants maintained similar water use efficiency levels regardless of nitrogen availability under non-stressed conditions. However, this pattern changed markedly under temperature stress, particularly in the low-nitrogen (LN) treatment, where _i_*WUE* was significantly lower compared to the medium- (MN) and high-nitrogen (HN) treatments at both 15 °C and 45 °C (Table 1). The maintenance of higher _i_*WUE* in the HN and MN treatments under temperature stress conditions suggests that adequate nitrogen supply enhances plant capacity to optimize water use under unfavorable temperatures. This nitrogen-mediated enhancement of water use efficiency under environmental stress aligns with previous observations in the literature [50]. The fact that plants with adequate nitrogen nutrition (HN and MN) maintained higher _i_*WUE* under both low- and high-temperature stress suggests that proper nitrogen management could be an effective strategy for improving plant water use efficiency under temperature stress conditions. This is particularly relevant in the context of extreme temperature fluctuations and water scarcity scenarios predicted under climate change.

It is pervasively believed that stomata play a crucial role in regulating the exchange of CO_2_ and H_2_O between the leaf interior and the atmosphere, thereby controlling the balance between photosynthetic carbon uptake and transpirational water loss [51,52]. The mechanisms underlying these responses can be elucidated by examining the coordination between photosynthetic rate (*A*) and stomatal conductance (*g*_s_), both of which are key components of intrinsic water use efficiency (_i_*WUE*). Under temperature stress, we observed that *g*_s_ showed significant differences among nitrogen treatments at 15 °C, but not at 45 °C (Table 1). This suggests that stomatal regulation plays a more important role in determining _i_*WUE* under low-temperature conditions. The lack of *g*_s_ response at the high temperature (45 °C) implies that other factors, such as biochemical processes, may become more dominant in controlling water use efficiency under heat stress [22,53]. Additionally, reduced mesophyll conductance under low N availability significantly limited photosynthesis at 15 °C, leading to decreased photosynthetic rates and _i_*WUE*, a phenomenon which suggests that mesophyll conductance played a crucial role in limiting photosynthesis under temperature stress, particularly in the LN treatment (Table 1). Thus, the reduced coordination between CO_2_ uptake and H_2_O loss through improved stomatal regulation and decreased mesophyll conductance are the main factors that determine _i_*WUE* under the interactive effect of nitrogen and temperature treatments. These findings contribute to our understanding of the physiological mechanisms underlying plant responses to temperature stress and highlight the importance of nitrogen nutrition in maintaining efficient water use under stressed conditions. Future research should focus on elucidating the molecular mechanisms governing the interaction between nitrogen status and temperature stress in regulating stomatal and mesophyll conductance, as well as their combined effects on plant water use strategies.

## 5. Conclusions

This study reveals distinct mechanisms of how temperature stress affects rice photosynthesis, with the low temperature (15 °C) showing stronger negative impacts than the high temperature (45 °C), primarily through increased mesophyll limitation. Increased nitrogen supply effectively mitigated temperature stress effects by maintaining better mesophyll conductance and photosynthetic efficiency. While photosynthetic nitrogen use efficiency (PNUE) decreased with increasing nitrogen supply under the optimal temperature (30 °C), the moderate nitrogen supply maintained optimal PNUE under temperature stress, suggesting that a balanced nitrogen level is crucial for maximizing both photosynthetic capacity and nitrogen use efficiency. Moreover, adequate nitrogen supplies enhanced intrinsic water use efficiency (_i_*WUE*) under both temperature stresses through improved coordination between CO_2_ uptake and water loss. These findings highlight the importance of optimizing nitrogen management for enhancing crop resilience to temperature extremes under climate change.

## Figures and Tables

**Table 1:** Interactive effects of nitrogen supply and temperature stress on leaf gas exchange parameters.

Treatment		*A* (μmol m^−2^ s^−1^)	*g*_s_ (mol m^−2^ s^−1^)	_i_*WUE* (μmol mol^−1^)	*E* (10^−3^ mol m^−2^ s^−1^)	*C*_i_ (μmol mol^−1^)	*g*_m_ (mol m^−2^ s^−1^)	*C*_c_ (μmol mol^−1^)	*ETR* (μmol m^−2^ s^−1^)
	HN	16.5 ± 0.7 de	0.46 ± 0.04 bcd	33.7 ± 4.5 ab	4.64 ± 0.4 d	319 ± 9 abc	0.09 ± 0.01 cd	142 ± 5 ab	152 ± 8 c
15 °C	MN	11.8 ± 1.5 fg	0.33 ± 0.03 cd	36.5 ± 7.8 ab	3.62 ± 0.3 d	324 ± 14 ab	0.06 ± 0.01 de	118 ± 8 cd	130 ± 9 cd
	LN	7.80 ± 1.2 g	0.28 ± 0.05 d	29.2 ± 9.2 ab	3.28 ± 0.5 d	339 ± 16 a	0.03 ± 0.01 e	92 ± 7 e	116 ± 9 d
	HN	30.8 ± 0.91 a	0.76 ± 0.21 a	42.7 ± 11 ab	11.6 ± 1.2 c	289 ± 18 bc	0.23 ± 0.02 a	155 ± 9 a	261 ± 7 a
30 °C	MN	24.0 ± 0.6 bc	0.45 ± 0.04 bcd	53.3 ± 5.5 a	9.06 ± 0.4 c	281 ± 8 c	0.16 ± 0.01 b	130 ± 3 bc	233 ± 6 ab
	LN	22.1 ± 2.6 bc	0.43 ± 0.03 bcd	51.5 ± 6.2 a	9.24 ± 0.8 c	287 ± 12 bc	0.14 ± 0.03 bc	123 ± 12 bcd	227 ± 12 b
	HN	25.8 ± 1.6 b	0.63 ± 0.14 ab	42.5 ± 7.4 ab	24.6 ± 0.7 a	278 ± 15 c	0.17 ± 0.00 b	124 ± 5 bcd	260 ± 7 a
45 °C	MN	20.4 ± 3.6 cd	0.49 ± 0.16 bcd	45.6 ± 18 ab	20.6 ± 3.9 b	280 ± 36 c	0.13 ± 0.05 bc	110 ± 10 de	233 ± 22 ab
	LN	15.3 ± 1.6 ef	0.57 ± 0.11 abc	27.5 ± 7.2 b	24.0 ± 1.8 ab	315 ± 15 abc	0.07 ± 0.01 de	94 ± 12 e	212 ± 25 b
T/N/T × N		***/***/ns	**/***/ns	*/ns/ns	***/**/ns	***/*/ns	***/***/ns	***/***/ns	***/***/ns

The following parameters were measured: photosynthetic rate (*A*); stomatal conductance (*g*_s_); intrinsic water use efficiency (_i_*WUE*); transpiration rate (*E*); intercellular CO_2_ concentration (*C*_i_); mesophyll conductance (*g*_m_); chloroplast CO_2_ concentration (*C*_c_); electron transport rate (*ETR*). Values are presented as means ± standard deviation (SD) with four biological replicates. Different letters indicate significant differences according to Tukey HSD (0.05). The significance levels for interactive effects are as follows: *, *p* < 0.05; **, *p* < 0.01; ***, *p* < 0.001; ns, not significant.

**Table 2:** The influence of varying nitrogen (N) supply levels on key agronomic characteristics.

Treatment	N_mass_ (mg/g)	N_area_ (g/m^2^)	LMA (g/m^2^)	Total Leaf Area (10^3^ cm^2^ Plant^−1^)	Tillers (No. Plant ^−1^)	Plant Height (cm)	Biomass (g plant^−1^)
HN	37.8 ± 1.3 a	2.13 ± 0.14 a	56.3 ± 4.7 a	1.79 ± 0.45 a	11.5 ± 1.7 a	114 ± 7 a	22.5 ± 6.9 a
MN	23.3 ± 0.8 b	1.27 ± 0.13 b	54.3 ± 4.1 a	0.77 ± 0.12 b	7.5 ± 0.6 b	96 ± 2 b	12.5 ± 0.5 b
LN	17.6 ± 0.43 c	1.02 ± 0.08 c	57.7 ± 3.3 a	0.28 ± 0.02 b	3.5 ± 0.6 c	84 ± 2 c	5.6 ± 0.5 b

The following parameters were assessed: leaf N content per unit dry weight (Nmass); leaf N content per unit area (Narea); leaf mass per area (LMA). Values are presented as means ± standard deviation (SD) with four biological replicates. Different letters indicate significant differences according to Tukey HSD (0.05).

## Data Availability

All data that support the results of this study are available from the corresponding author upon reasonable request.

## Supplementary Materials

The following supporting information can be downloaded at: https://www.mdpi.com/article/10.3390/plants14060961/s1, Figure S1: Positive correlations between diffusional conductance (*g*_s_: stomatal conductance; *g*_m_: mesophyll conductance) and photosynthetic rate (*A*), as well as between biochemistry (*V*_c,max_: maximum Rubisco carboxylation capacity; *J*_max_: maximum electron transport rate) and photosynthetic rate (*A*); Table S1: Interactive effects of nitrogen supply and temperature stress on leaf potential photosynthetic capacity.
