# Supplementary material for: Nitrogen Supply Mitigates Temperature Stress Effects on Rice Photosynthetic Nitrogen Use Efficiency and Water Relations
Source: Plants (Basel). 2025 Mar 19;14(6):961. doi: 10.3390/plants14060961 (PMC11945697; doi:10.3390/plants14060961)
Supplement: Supplementary file 1 [file plants-14-00961-s001.zip › plants-3495579-Supplementary Materials-main.pdf]

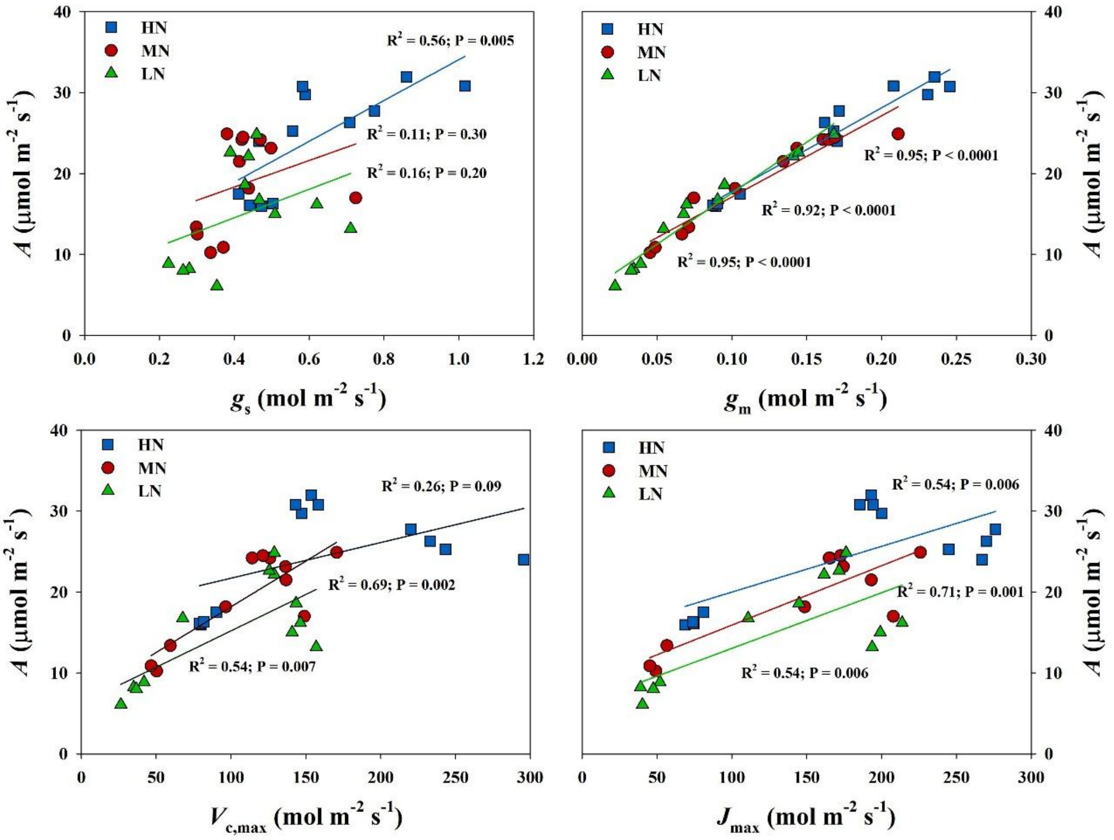

Figure S1. Positive correlations between diffusional conductance (g_s: stomatal conductance; g_m: mesophyll conductance) and photosynthetic rate (A), as well as between biochemistry ($V_{c,max}$: maximum Rubisco carboxylation capacity; J_{max}: maximum electron transport rate) and photosynthetic rate (A).

Table S1. Interactive effects of nitrogen supply and temperature stress on leaf potential photosynthetic capacity.

Treatment		$V_{c,max}$ (μmol m^{-2} s^{-1})	J_{max} (μmol m^{-2} s^{-1})	*TPU* (μmol m^{-2} s^{-1})
15 °C	HN	82.7 ± 5.2 cd	74.6 ± 5.0 c	5.0 ± 0.4 d
	MN	52.2 ± 6.6 d	50.3 ± 5.8 c	3.6 ± 0.4 d
	LN	35.0 ± 6.4 d	44.7 ± 6.1 c	3.0 ± 0.4 d
30 °C	HN	150.5 ± 6.8 b	193.3 ± 6.0 b	11.9 ± 0.4 c
	MN	124.5 ± 9.3 bc	169.5 ± 4.8 b	10.9 ± 0.4 c
	LN	131.7 ± 8.0 bc	163.6 ± 13.9 b	11.1 ± 0.9 c
45 °C	HN	248.0 ± 33.1 a	264.5 ± 13.6 a	15.9 ± 0.8 a
	MN	138.2 ± 31.1 b	193.9 ± 33.0 b	14.4 ± 2.0 ab
	LN	128.0 ± 40.1 bc	179.4 ± 46.5 b	12.5 ± 1.6 bc
T/N/T × N		***/***/**	***/***/*	***/***/ns

The following parameters were analyzed: maximum Rubisco carboxylation capacity ($V_{c,max}$); maximum electron transport rate (J_{max}); triose phosphate utilization rate (TPU). Values are presented as means ± standard deviation (SD) with four biological replicates. Different letters indicate significant differences according to Tukey HSD (0.05). Statistical significance of interactive effects: *, $p < 0.05$; **, $p < 0.01$; ***, $p < 0.001$; ns, no significant.
